# Artificial intelligence-generated encounter summaries: early insights from ambulatory clinicians at a large academic health system

**DOI:** 10.1093/jamiaopen/ooaf096

**Published:** 2025-09-02

**Authors:** Jared Silberlust, Priyanka Solanki, Elizabeth R Stevens, Nicholas Genes, Edward Lim, Kevin Sun, Marisa Lewis, Paul Testa, Adam Szerencsy

**Affiliations:** MCIT Department of Health Informatics, NYU Langone Health, New York, NY 10016, United States; MCIT Department of Health Informatics, NYU Langone Health, New York, NY 10016, United States; MCIT Department of Health Informatics, NYU Langone Health, New York, NY 10016, United States; MCIT Department of Health Informatics, NYU Langone Health, New York, NY 10016, United States; MCIT Clinical Systems, NYU Langone Health, New York, NY 10016, United States; MCIT Clinical Systems, NYU Langone Health, New York, NY 10016, United States; MCIT Clinical Systems, NYU Langone Health, New York, NY 10016, United States; MCIT Department of Health Informatics, NYU Langone Health, New York, NY 10016, United States; MCIT Department of Health Informatics, NYU Langone Health, New York, NY 10016, United States

**Keywords:** artificial intelligence, chart review, chart summarization, provider efficiency

## Abstract

**Objectives:**

Assess time impact and provider perception of AI-generated encounter summaries.

**Materials and Methods:**

An artificial intelligence (AI) clinical note summarization tool was deployed in ambulatory practices for 22 providers. A 6-month pre-post analysis evaluated changes in EHR time, and survey feedback assessed tool utility.

**Results:**

Among 17 providers with complete Epic Signal time data, average time spent in clinical review per visit decreased from 3:22 minutes pre-intervention (8345 visits) to 3:04 minutes post-intervention (7924 visits). Higher baseline review time was significantly associated with greater post-implementation reductions, with each additional minute at baseline linked to an 8.6-second decrease in review time (*P* = .046). The majority of providers reported high tool accuracy and time saving.

**Discussion:**

AI-generated summaries appear most beneficial for clinicians who initially spend the longest time in chart review, suggesting a targeted efficiency gain. Providers expressed optimism about the tool’s potential to streamline clinical workflows.

**Conclusion:**

Early experience with AI-generated summaries shows promise in improving provider workflow.

## Background and significance

Healthcare providers spend considerable time reviewing patient charts before appointments, with estimates approaching 80 minutes per day for ambulatory clinicians.^1^ The burden of chart review contributes to burnout and dissatisfaction, especially when review workflows are inefficient or poorly supported by technology.^2–4^ Recent advances in artificial intelligence (AI), particularly large language models, have enabled new forms of clinical decision support (CDS), including automated summarization of prior clinical notes.^5–7^ While these tools have the potential to reduce review time and improve information synthesis, their real-world value depends on trust, usability, and meaningful workflow integration. Moreover, it remains unclear which subgroups of providers may benefit most from such interventions. This case report is, to our knowledge, the first to evaluate the impact of an AI-generated note summarization tool implemented in ambulatory clinics across a large academic health system, using a combination of time-based metrics and clinician feedback.

## Objectives

The objectives of this work are to (1) assess how AI-generated encounter summaries affect time spent in chart review, (2) identify whether certain providers may benefit more than others from AI summarization tools, and (3) evaluate user perceptions of an AI-generated encounter summary tool on accuracy, usefulness, and integration into daily workflow.

## Methods

**Figure 1.**
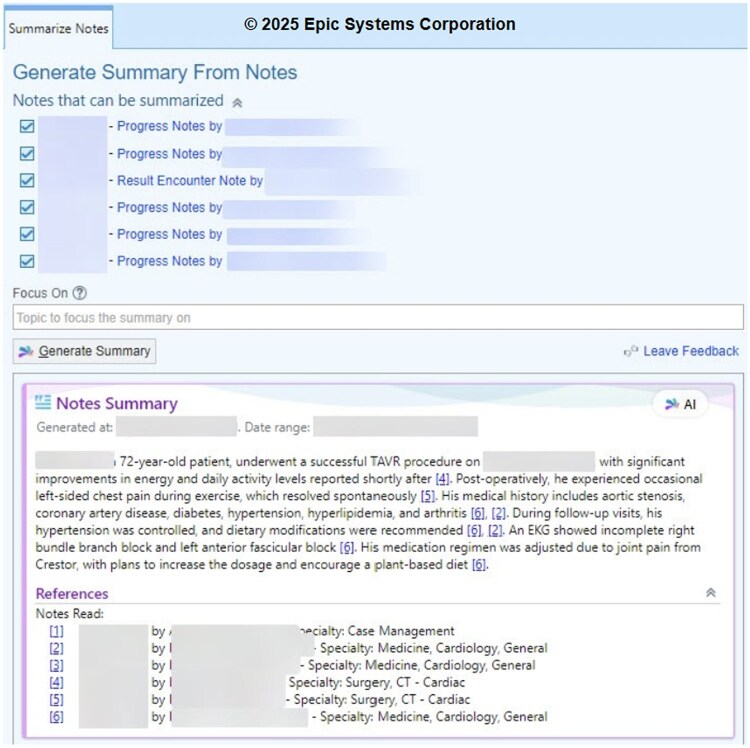
AI encounter summary tool.

The intervention took place at NYU Langone Health, an academic health system using the Epic electronic health record (EHR). The EHR-vendor-developed AI note summarization tool was implemented across several outpatient practices in November 2024 with a pilot group of 22 clinicians from 9 different specialties. This group was chosen based on prior participation in digital health initiatives and volunteered to participate in this implementation effort, reflecting a population likely to be more receptive to new technology. The summarization tool used GPT-4 via OpenAI’s API, as incorporated into the Epic EHR platform, to generate brief summaries from existing notes in the patient’s chart (Figure 1). While the specific version (eg, GPT-4o) was not directly accessible to end users, Epic documentation at the time of implementation indicated the model was consistent with GPT-4-turbo. These summaries appeared in the Clinical Review activity within Epic and were displayed upon opening the chart prior to each patient visit.

Three sources of data were analyzed for this case report. First, analytics for time spent in chart review (Epic Signal^8^) were collected for the three months before and after implementation. Pre-implementation time was measured from August to October 2024; post-implementation from December 2024 to February 2025. To assess differential impact, providers were pre-specified into two groups based on their initial average chart review time, using the institutional average of 3.4 minutes per visit as the cutoff; those above this threshold were categorized as higher-time reviewers and those at or below as lower-time reviewers. A Wilcoxon signed-rank test was performed to determine change in time spent in chart review per appointment pre- and post-intervention for all physicians, and a Mann-Whitney *U* test was used to compare change in time spent between physicians grouped by initial speed of chart review, high and low. To further evaluate whether baseline chart review time predicted post-implementation changes, we computed the change in mean review time for each physician (post minus pre) and performed a simple linear regression with baseline review time as the independent variable and change in review time as the dependent variable.

Second, a real-time feedback mechanism within the tool allowed users optionally to provide feedback on the summary with one or multiple of the following: “The summary was useful and accurate,” “The summary was too long or included irrelevant information,” “The summary was too short or missed important information,” “The summary had inaccurate or made-up information,” “The summary was difficult to follow or confusing,” “The summary took too long to generate,” and “The references did not match the information in the summary.” The total response rate of each of these response options was recorded.

Third, all users were surveyed after 1 month of using the tool. The survey included a 5-point Likert scale with choices from Poor to Excellent, asking users to rate the tool for usefulness, accuracy, completeness, safety, and relevance. It also included a second Likert scale question, from Strongly Disagree to Strongly Agree, asking clinicians to rate the tool’s ability to save them time, improve their efficiency, inform them of details about their patients, and whether they would prefer to work for a practice that made this tool available. Finally, it asked clinicians to select whether the AI summarization had informed them of new information about their patients, if they referenced information directly from the summary in their clinical encounter, if they included the summarized text in their notes, if they have incorporated using the tool in their pre-visit planning, and if they skipped reviewing full-length notes because of the tool’s output. This project met Institutional Review Board (IRB) criteria for quality improvement, and IRB review and informed consent were waived. Data analysis was performed using Python version 3.10 (GCC).

## Results

**Figure 2.**
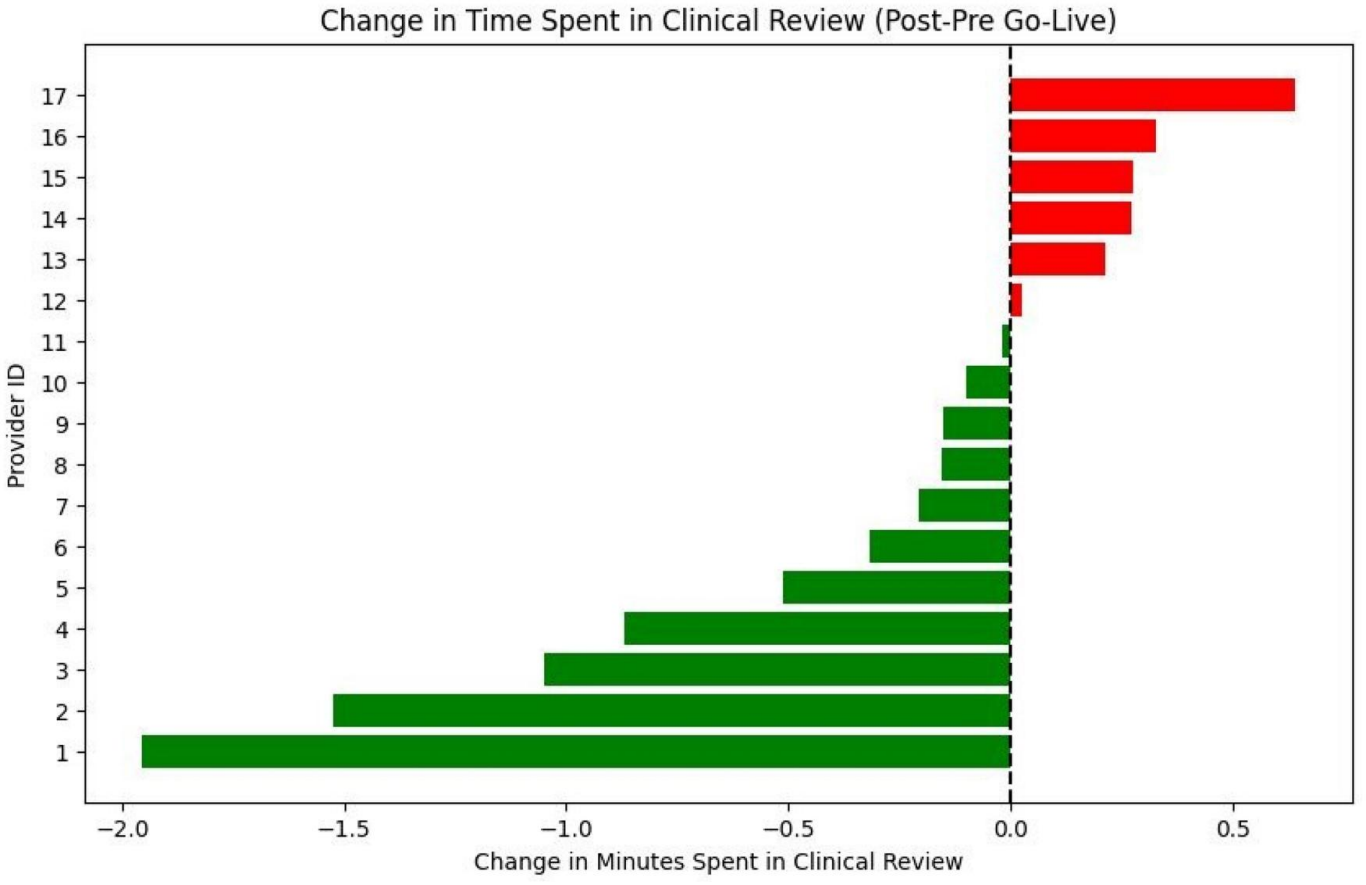
Change in minutes spent in clinical review per patient after implementation of the AI encounter summarization tool. Red indicates increased time and green indicates decreased time after implementation.

Of the 22 participating clinicians, 5 were cardiologists, 4 were internists, 2 were gastroenterologists, 2 were obstetrician/gynecologists, 2 were pediatricians, 2 were radiologists, 1 was a hematologist/oncologist, 1 was a palliative care specialist, 1 was a nephrologist, 1 was a medicine-pediatric dual-certified physician, and 1 was a general surgery clinician. 17 of the 22 clinicians had complete Epic Signal data for each month during the study period. 5 providers were excluded due to missing data resulting from low patient volumes below Epic’s reporting threshold in at least one of the six reporting months—to be included, a provider needed at least 5 appointments scheduled per week within the entire reporting period. Across the 17 providers, the average time spent in clinical review per visit decreased from 3:22 minutes in 8345 visits pre-intervention to 3:04 minutes in 7924 visits post-intervention. Eleven providers had an average decrease in time spent in clinical review per visit, and 6 providers had an average increase (Figure 2).

**Figure 3.**
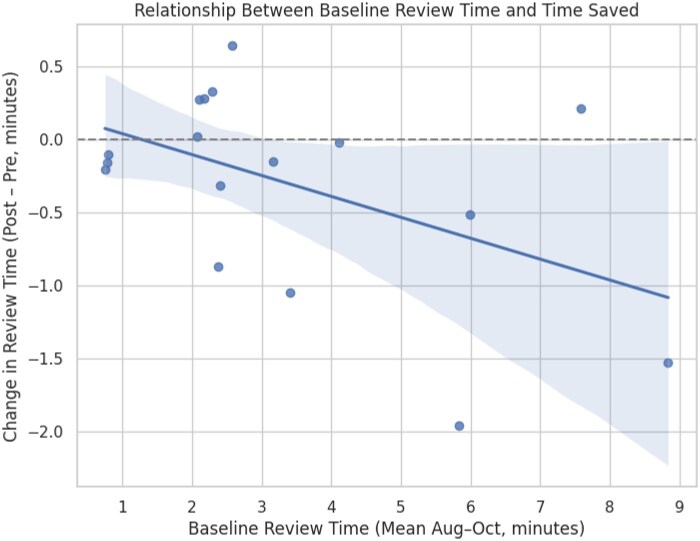
Association between baseline review time and change in review time post-implementation. Each point represents a physician. The shaded area shows the 95% confidence interval for the regression line.

A Wilcoxon signed-rank test comparing the time spent in clinical review pre- and post-intervention for the entire cohort of 22 clinicians yielded a test statistic of 50.0 (*P* = .22), indicating no statistically significant difference overall. The “longer-initial-time” group (*n* = 6 providers with >3.4 minutes pre-intervention) showed a 49-second reduction from 5:58 minutes per visit across 2045 visits to 5:09 minutes per visit across 2050 visits, while the “shorter-initial-time” group (*n* = 11 providers with <3.4 minutes pre-intervention) reduced 1 second from 1:57 minutes per visit across 6300 visits to 1:56 minutes per visit across 5874 visits. A Mann-Whitney *U* test comparing the magnitude of change between groups yielded a U statistic of 14.0 (*P* = .03), demonstrating a statistically significant improvement among longer-time clinicians relative to lower-time clinicians. Subsequent linear regression revealed that baseline review time was significantly associated with reduction in review time post-intervention (β = –0.143, *P* = .046), indicating that for every additional minute spent at baseline, physicians reduced their review time by an average of 0.14 minutes (8.6 seconds). This model explained 24% of the variance in time saved (*R*^2^ = 0.24). A scatter plot confirmed this negative association, with physicians who had higher initial review times experiencing greater reductions (Figure 3).

Next, the optional real-time feedback tool was used by 16 providers in 444 of the patient summaries post go-live. Seven providers elected not to leave real-time feedback. Given providers were able to select multiple feedback options for each summary, 507 unique feedback entries were collected for the 444 patients. The results are summarized in Table 1.

**Table 1.** Real-time feedback results of the AI encounter summary tool.

Real-time feedback option	Number of summaries (% of total) (*n* = 444)
The summary was useful and accurate.	269 (60.6%)
The summary was too short or missed important information.	86 (19.3%)
The summary was too long or included irrelevant information.	80 (18.0%)
The summary took too long to generate.	44 (9.9%)
The summary was difficult to follow or confusing.	15 (3.4%)
The summary had inaccurate or made-up information.	13 (2.9%)
The references did not match the information in the summary.	0 (0.0%)

Finally, all 22 providers completed the survey 1 month after starting to use the tool. The survey results are summarized in Table 2.

**Table 2.** 1-month post AI encounter summary implementation survey results (*n* = 22).

AI encounter summary survey item	Providers rating good, very good, or excellent (%)
Overall usefulness	15 (68.2%)
Accuracy	19 (86.4%)
Completeness	13 (59.1%)
Safety	21 (95.5%)
Relevance	16 (72.8%)

	Providers rating agree or strongly agree (%)
The tool has saved me time reviewing prior notes before a visit.	12 (54.6%)
The tool has helped me become more efficient when preparing for visits.	7 (31.8%)
I am better informed about my patients’ care prior to a visit when using the tool.	12 (54.6%)
I would prefer to work for a practice that provides an AI-powered encounter summarization tool.	14 (63.7%)

	Providers selecting the listed option (%)
I have learned new information about my patients from the summarization tool.	14 (63.6%)
I have referenced it during a patient encounter to guide the discussion	5 (22.7%)
I have included text from the summaries in my notes.	4 (18.2%)
I have incorporated it into my pre-visit planning	12 (54.6%)
I have skipped reviewing full-length notes because the summary provided enough context	5 (22.7%)
None of the above	2 (9.1%)

## Discussion

This case report describes the early implementation of an AI-powered, EHR-integrated clinical summarization tool and its impact on provider experience in ambulatory care. While the overall reduction in clinical review time was modest—an average of 18 seconds per visit—it was not statistically significant across the full cohort. However, a meaningful trend emerged among clinicians who initially spent more than 3.4 minutes per visit reviewing charts. These providers saw a statistically significant reduction of 49 seconds per visit, and regression analysis showed that each additional minute spent at baseline predicted an 8.6-second time savings. This suggests that such tools may be most effective when targeted to providers with greater documentation demands or those managing complex patients.

User feedback aligned with this finding. Most clinicians rated the tool as accurate (86%), useful (68%), and safe (95%). Over half reported that the summaries improved pre-visit understanding (55%) and saved time (55%). Additionally, 64% indicated they would prefer to work at a practice offering such a tool. These findings support the tool’s perceived utility and potential to enhance provider satisfaction when appropriately integrated.

However, experiences varied. Only 32% of clinicians reported improved overall efficiency, and fewer incorporated the summaries into documentation (18%) or discussions with patients (23%). These findings may reflect differences in trust, workflow compatibility, or summary quality. Notably, nearly 10% of real-time feedback responses indicated that the summary took too long to generate—likely occurring when users regenerated content based on different note selections. Because summary generation time was not captured in EHR logs, this represents a limitation in our measurement strategy and highlights the need for future tools to log generation latency.

Perceived inaccuracies also shaped user experience. Of 444 real-time feedback entries, 13 (2.9%) flagged hallucinations or factual errors. All were reviewed by institutional informatics leadership, Epic engineers, and clinicians. These hallucination examples included summaries that listed an incorrect date for a prior endoscopy and stated that a patient was not on anticoagulation treatment when they actually were. While these were marked as potentially dangerous for patient care, several other reported inaccuracies were due to user misinterpretation rather than true model error—such as summaries reflecting accurate information that clinicians initially overlooked. These instances underscore the need for clinician verification and raise the broader question of what level of AI inaccuracy is acceptable in clinical workflows.

Importantly, 22.7% of providers reported sometimes skipping full-length notes in favor of the summary alone. While this may reflect confidence in the tool, it also raises the growing concern of human, data, and algorithmic biases in the implementation of these AI tools. These biases can directly result in inaccurate information used in the provider’s clinical reasoning process as well as scaling the tool to all outpatient clinical settings. However, throughout the life cycle of an AI tool, biases can be mitigated from the conception phase to post-deployment.^9^ We suggest that organizations that implement AI summarization tools consider safeguards, such as uncertainty highlighting, monitoring usage patterns, and reinforcing clinical judgment, to prevent over-reliance on summarization alone. The development of rigorous, comprehensive standards for the use of AI summaries in medicine is necessary to ensure patient safety without compromising clinical efficiency.^10^

Interestingly, not all users benefited. Six clinicians experienced an increase in chart review time post-implementation. Several explanations are possible: cognitive friction from evaluating AI output, delays in summary availability, or a mismatch between summary content and individual workflow needs. This finding highlights the importance of tailoring implementation and underscores the need for future qualitative research to understand barriers to adoption.

Together, these findings reinforce several principles critical to the successful implementation of Generative AI to facilitate patient care. First, AI tools should be embedded into existing workflows with minimal friction,^11^ as was done directly within the EHR’s Clinical Review activity. Second, real-time user feedback is essential to iteratively improve performance and build trust.^12^^,^^13^ While we were not able to directly update the prompt to optimize the output of the summary, regular discussions with the vendor were part of the iterative process. Third, a “one-size-fits-all” approach may dilute perceived benefit^14^; tailoring interventions based on provider characteristics or workflows—such as prioritizing deployment for higher-time reviewers—may enhance impact.

This work also demonstrates the value of combining system-generated performance data (Epic Signal) with clinician-reported feedback to evaluate CDS interventions. Signal provides an objective view of time use, while survey and feedback mechanisms capture human experience—both are essential dimensions in the sociotechnical model of health IT.^15^^,^^16^

This study has several limitations. The sample size was small, with limited specialty representation and a short observation period. Providers were selected based on prior engagement in digital health initiatives and volunteered to participate, introducing potential selection bias. The cohort may not reflect broader clinical populations with varying levels of digital comfort. We also did not capture provider seniority or years in practice, limiting our ability to assess how experience level may influence adoption or perception. Anecdotally, no clear patterns emerged by seniority, but this warrants further study.

Our subgroup analysis, while pre-specified based on institutional Signal data, is subject to potential regression to the mean. Without a control group, we cannot rule out secular trends or unmeasured confounders. Future studies should incorporate matched control groups using broader Signal data to better assess causality.

Looking ahead, further research should explore the long-term effects of summarization tools on efficiency, documentation quality, patient outcomes, and clinician burnout. Qualitative studies could also help elucidate how summary content influences diagnostic reasoning and decision-making and how clinicians perceive and mitigate AI inaccuracies. Organizations deploying such tools must remain vigilant in monitoring performance, ensuring responsible use, and maintaining clinician oversight.

In summary, this early evaluation suggests that AI-generated encounter summaries hold promise for improving pre-visit preparation, particularly for clinicians with high documentation burdens. With thoughtful implementation and ongoing refinement, these tools can help augment—rather than replace—clinical judgment in everyday care.

## Data Availability

The data underlying this article will be shared on reasonable request to the corresponding author.
